# Long-term prospective outcome data using EndoPredict as risk stratification and chemotherapy decision biomarker in hormone receptor-positive, HER2-negative early breast cancer

**DOI:** 10.1007/s10549-024-07346-2

**Published:** 2024-05-09

**Authors:** Evelyn Klein, Marion Kiechle, Adriana Josipovic, Sophie-Isabelle Anders, Aurelia Noske, Carolin Mogler, Alexander Hapfelmeier, Johannes Ettl

**Affiliations:** 1 Department of Obstetrics and Gynecology, Klinikum rechts der Isar, School of Medicine and Health, Technische Universität München, Ismaninger Str. 22, 81675 Munich, Germany; 2 Klinik für Geburtsmedizin, Campus Charité Mitte, Charité - Universitätsmedizin, Berlin, Germany; 3 Institute of Pathology, Klinikum rechts der Isar, School of Medicine and Health, Technische Universität München, Munich, Germany; 4 Institute of AI and Informatics in Medicine, School of Medicine and Health, Technische Universität München, Munich, Germany; 5 Klinikverbund Allgäu, Frauenheilkunde und Geburtshilfe, Klinikum Kempten, Kempten, Germany; 6 Institute of General Practice and Health Services ResearchSchool of Medicine and Health, Technische Universität München, Munich, Germany

**Keywords:** Breast cancer, Prognostic biomarker, Predictive biomarker, EndoPredict, Adjuvant chemotherapy, Risk stratification

## Abstract

**Purpose:**

To report the prospective long-term outcome data of patients whose chemotherapy decision was guided by the EndoPredict test.

**Methods:**

Patients with hormone receptor-positive HER2-negative early breast cancer with 0–3 positive lymph nodes were enrolled. The EndoPredict test was carried out on all tumor samples. Treatment compliance, local recurrence, distant metastases, and survival were evaluated. Associations of EPclin risk stratification with 5-year disease-free survival and distant metastasis-free survival were evaluated by time-to-event analysis.

**Results:**

368 consecutive patients were included in the analysis. Median follow-up was 8.2 years. EndoPredict allocated 238 (65%) in the low-risk and 130 (35%) patients in the high-risk group. Risk for disease recurrence or death in EPclin high-risk patients was twofold higher than in EPclin low-risk patients (hazard ratio [HR] 2.08; 95% CI 1.26–3.44; *p* = 0.004). EPclin low-risk patients had a 5-year disease-free survival of 95.3% (95% CI 92.6–98.0%). EPclin high-risk patients were at higher risk of developing distant metastases or death (HR 2.21; 95% CI 1.27–3.88; *p* = 0.005). EPclin high-risk patients who underwent chemotherapy had a 5-year DFS of 89.1% (95% CI 82.7–96.1%) in contrast to high-risk patients without chemotherapy (68.9%; 95% CI 56.2–84.5%; HR 0.46; 95% CI 0.23–0.95; *p* = 0.036). EPclin high-risk patients were at higher risk of experiencing distant metastases or death than EPclin low-risk patients regardless of menopausal status (premenopausal: HR 3.55; 95% CI 1.17–12.32; *p* = 0.025; postmenopausal: HR 1.92; 95% CI 0.99–3.7; *p* = 0.054).

**Conclusion:**

EndoPredict can guide decisions on adjuvant chemotherapy in early luminal breast cancer. EndoPredict risk stratification is also applicable in premenopausal women.

## Introduction

Endocrine therapy is the mainstay treatment for patients with hormone receptor-positive, human epidermal growth factor receptor 2 (HER2)-negative, early breast cancer. Although endocrine therapy has been shown to reduce the risk of recurrence and improve survival, the lifetime risk of recurrence remains high. International guidelines recommend that patients at high risk of recurrence receive neoadjuvant or adjuvant chemotherapy in addition to endocrine therapy, while those at low risk may be spared chemotherapy [1].

In addition to clinicopathological factors such as tumor size, tumor grade, and tumor proliferation that have been traditionally used to determine the individual risk of recurrence, several genomic tests have been developed in the last two decades to predict patients’ prognosis and response to chemotherapy, in order to guide decisions on adjuvant endocrine and chemotherapy decisions in early breast cancer [1, 2]. Validated and commercially available genomic tests include Oncotype DX, MammaPrint, Prosigna, Breast Cancer Index, and EndoPredict [3]. EndoPredict test is an RNA-based 12-gene expression assay that measures the expression of three proliferative and five estrogen receptor (ER) signaling-associated genes, together with four normalization and control genes, by quantitative real-time polymerase chain reaction. Unlike some tests that need to be centrally determined, the EndoPredict test can be carried out on formalin-fixed paraffin-embedded (FFPE) tumor tissue in local laboratories [4]. The12-gene molecular score is combined with tumor size and nodal status to produce the EPclin score.

The prognostic value of EndoPredict has been validated in several prospective-retrospective trials [3, 5–10]. Recently, the prognostic power of EndoPredict was also shown in a study with premenopausal patients [11]. In addition, a retrospective comparative analysis of data on 3746 women enrolled in clinical trials who received chemotherapy plus endocrine therapy versus endocrine therapy alone showed that a high EPclin score predicted chemotherapy benefit in ER-positive HER2-negative disease [12]. As most studies have been conducted in postmenopausal women, the American Society of Clinical Oncology (ASCO) guidelines currently recommend the use of EndoPredict to guide treatment decision only in postmenopausal women with node-negative or node-positive with 1–3 positive nodes, hormone receptor-positive HER2-negative early breast cancer [2].

We previously reported the first prospectively collected outcome data of 373 patients whose adjuvant systemic therapy recommendation was based on the EPclin risk classification in clinical routine [13]. The initial report after 3 years of follow-up showed a twofold risk of disease recurrence or death in EPclin high-risk patients compared with EPclin low-risk patients (hazard ratio [HR] 2.05; 95% confidence interval [CI] 0.85–4.96; *p* = 0.110) and a significant higher risk of distant metastases (HR 5.18; 95% CI 1.04–25.74; *p* = 0.044). Among the EPclin high-risk patients, those who underwent adjuvant chemotherapy were at lower risk for death or recurrence than those who did not receive adjuvant chemotherapy (HR 0.32; 95% CI 0.10–1.05; *p* = 0.061). The EPclin high-risk patients who received standard adjuvant chemotherapy experienced a 68% reduction in relapse compared to those who decided not to undergo the recommended chemotherapy.

In this article, we report an updated analysis of the cohort after a median follow-up of 8.2 years. We also evaluated EPclin-based risk stratification in correlation to clinicopathological factors and in premenopausal patients.

## Patients and methods

### Study population

Patients with ER-positive, HER2-negative early breast cancer with 0–3 positive lymph nodes were enrolled at the interdisciplinary breast center of Klinikum rechts der Isar, Technical University Munich, Germany, between March 2012 and 2015. The EndoPredict test was carried out on all tumor samples. Demographic, clinical, and pathological data were assessed for each patient at baseline. All patients underwent curative surgery. Therapy recommendations were given for all patients during an interdisciplinary tumor board discussing each case individually. Endocrine therapy was advised in every case and decision for or against chemotherapy was primarily based on the EPclin risk classification, taking individual comorbidity into account. In every case, the recommended as well as the performed treatments were documented. Follow-up for each patient was recorded including treatment compliance, local recurrence, distant metastases, and survival.

### EndoPredict analyses

EndoPredict assays (Myriad International GmbH, Cologne, Germany) were performed on FFPE tissue samples of primary breast tumors at the Institute of Pathology at Klinikum rechts des Isar, Technical University Munich, according to the manufacturer’s instructions, as described previously [4]. The validated cut-off value of 3.3 for the EPclin score was used for risk discrimination [5]. Patients with an estimated risk of distant recurrence of more or equal to 10% at 10 years were categorized as high-risk.

### Ki-67 analysis

Immunohistochemically determined Ki-67 staining was used to distinguish between the ‘luminal A’ and ‘luminal B’ biological BC subtypes. Immunohistochemical staining of Ki-67 was carried out on whole slide sections of archival breast cancer resection specimens. Briefly, after deparaffinization and antigen demasking, the slides were incubated with the primary antibody against ki-67 (clone MIB1, 1:50, DAKO 7240, Denmark) on an automated staining system (BenchMark XT, Ventana Tucson, AZ). Antibody binding was visualized using DAB as chromogen. Ki-67 scoring was performed according to the recommendations from the International Ki-67 in Breast Cancer Working Group [14]. A board-certified pathologist specialized in breast cancer performed the analysis, blinded without knowledge of the EndoPredict test results. In order to preclude inter-observer variability, all ki-67 evaluations were performed by the same pathologist.

### Statistical analysis

Survival analysis is reported using 5-year disease-free survival (DFS) as the primary time-to-event endpoint. DFS was defined as time to any recurrence (local, locoregional, or distant) or death by any cause (with or without recurrence), which ever occurred first. Distant metastasis-free survival (DMFS) was defined as time to any distant recurrence or death by any cause (with or without recurrence), which ever occurred first. We also analyzed tumor-related metastasis-free survival. Tumor-related metastasis-free survival was defined as the time to distant recurrence or cancer-related death, which ever occurred first. Risk estimates were obtained by the Kaplan–Meier method and cumulative risk functions in case of competing risks. Group comparisons were performed by Cox proportional hazards regression models and quantified through HRs. Median follow-up was estimated by the inverse Kaplan–Meier method. Spearman’s correlation coefficients were computed to quantify the bivariate relation of quantitative variables. Exploratory hypothesis testing was conducted at two-sided 5% significance levels.

## Results

### Study population

Of the 373 consecutive cases that were enrolled, five were excluded from subsequent analyses due to missing data. The current analysis was therefore performed in 368 patients. Patient and tumor characteristics are shown in Table 1. EndoPredict allocated 238 patients (64.7%) in the EPclin low-risk and 130 patients (35.3%) in the EPclin high-risk group. The proportion of premenopausal and postmenopausal patients within each risk category was similar. For the 362 female patients (98.4%), the distribution of EPclin score was as follows: 32.9% (*n* = 77) premenopausal patients and 67.1% (*n* = 157) postmenopausal patients in the EPclin low-risk group (*n* = 234), and 35.2% (*n* = 45) premenopausal and 64.8% (*n* = 83) postmenopausal patients in the EPclin high-risk group (*n* = 128).

**Table 1:** Patient characteristics

Characteristics	
Sex	
Female	362 (98.4)
Male	6 (1.6)
Age	
	60 (± 12.2)
Menopausal status	
Premenopausal	122 (33.2)
Postmenopausal	240 (65.2)
Histologic subtype	
Invasive ductal (NST)	259 (70.4)
Invasive lobular	70 (19.0)
Others	37 (10.1)
Tumor size	
pT1a	20 (5.4)
pT1b	63 (17.1)
pT1c	145 (39.4)
pT2	129 (34.8)
pT3	12 (3.3)
Nodal status	
Nodal negative	280 (76.1)
Nodal positive (1–3 lymph nodes)	88 (23.9)
Grading	
G1	70 (19.0)
G2	238 (64.7)
G3	60 (16.3)
Ki-67	
< 10%	73 (23.9)
10–25%	170 (55.6)
> 25%	63 (20.6)
Chemotherapy	
No	278 (75.5)
Yes	90 (24.5)
EPclin score	
≤ 3.3 (low risk)	238 (64.7)
> 3.3 (high risk)	130 (35.3)

Numbers are *n* (%) or mean ± SD

### Therapy recommendation and compliance

**Fig. 1:**
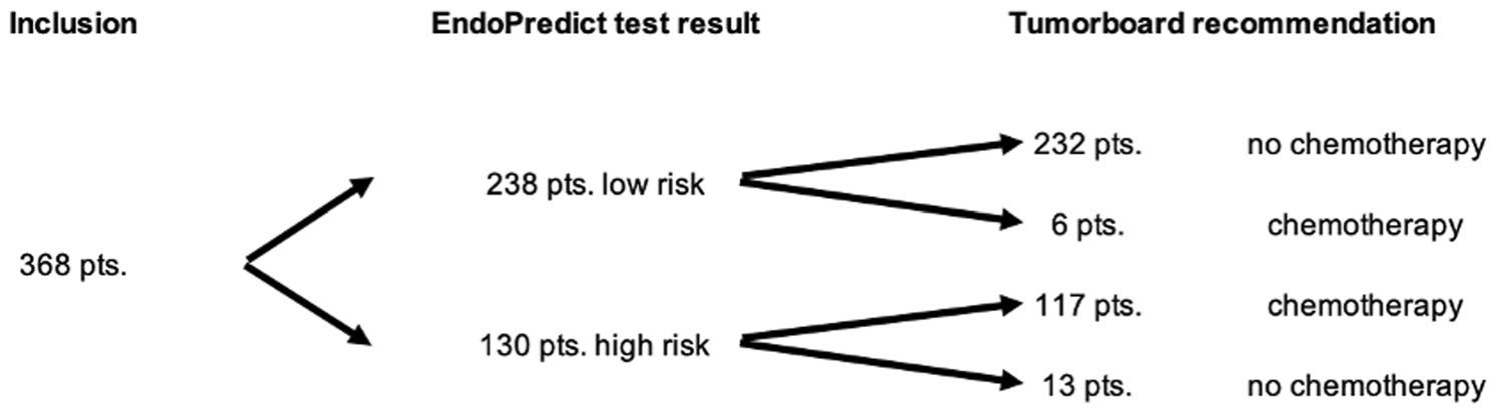
Patients’ EndoPredict test results and final tumor board recommendations

The tumor board recommendations for adjuvant chemotherapy according to EndoPredict test results are shown in Fig. 1. Six of the 238 EPclin low-risk patients (2.5%) were recommended to undergo chemotherapy despite their low-risk results, due to young age, multicentric tumor, or contralateral breast cancer. On the other hand, 13 of the 130 EPclin high-risk patients (10.0%) were recommended not to undergo chemotherapy despite their high-risk results, based on their individual risk factors such as age or comorbidities. Patients who were not recommended to undergo chemotherapy despite being EPclin high-risk were more likely to be older, have G1 tumors, and less likely to have nodal involvement (Table 2).

**Table 2:** Clinicopathological factors in the EPclin high-risk patients according to chemotherapy recommendation and compliance

High risk patients	Patients (*n*)	Age (mean)	EPclin (mean)	N+ (%)	G1 (%)	G3 (%)
Chemotherapy recommendation, non-compliant	30	64.2	4	14 (46.7%)	3 (10%)	8 (26.7%)
Chemotherapy recommendation, compliant	87	56.6	4	50 (57.5%)	4 (4.6%)	30 (34.5%)
No chemotherapy recommendation	13	65.1	3.5	6 (46.2%)	3 (23.1%)	5 (38.5%)

**Fig. 2:**
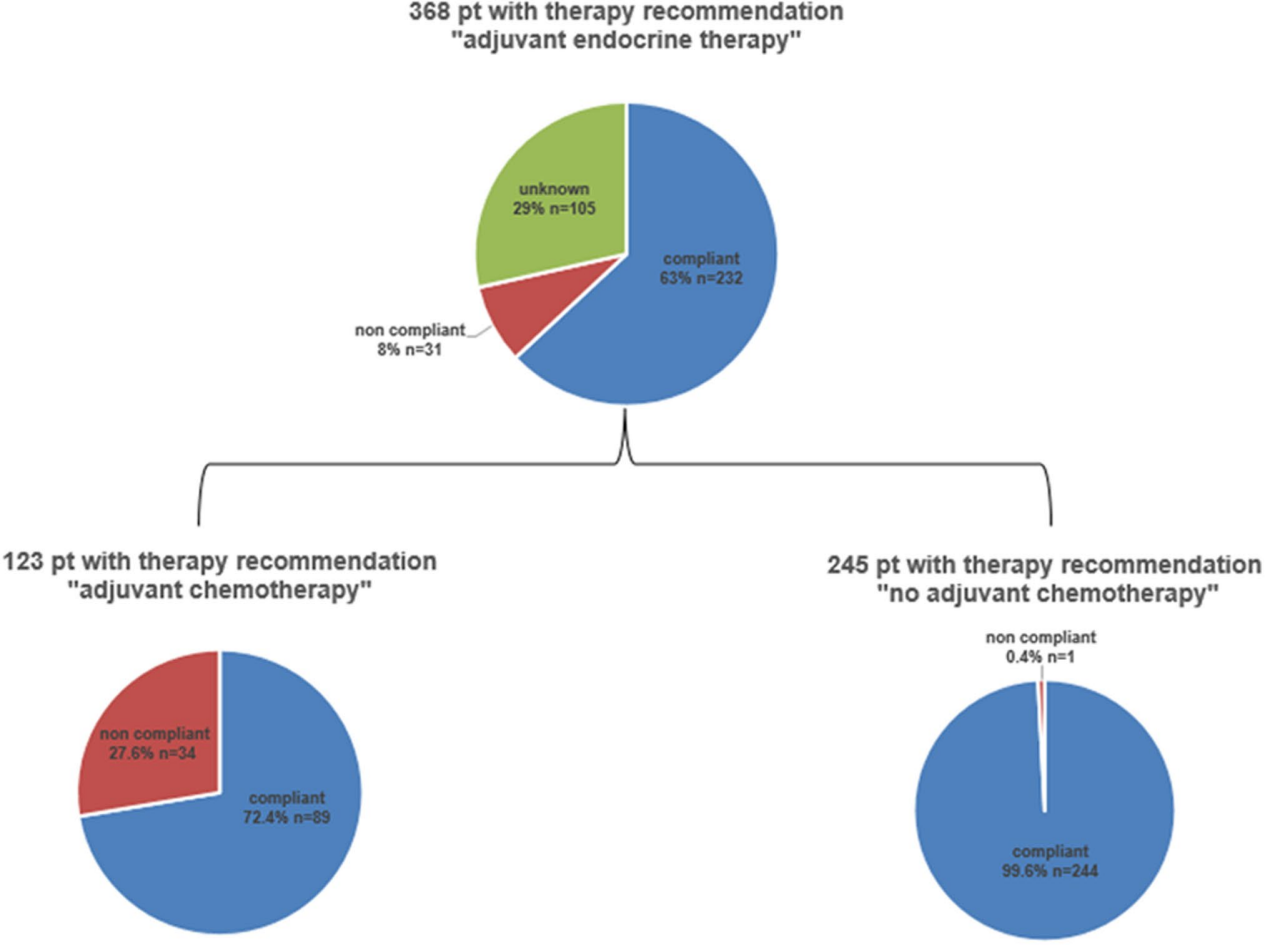
Treatment recommendations given by the tumor board and patients’ compliance

The treatment compliance is shown in Fig. 2. Adjuvant endocrine therapy for at least 5 years was recommended to all patients (*n* = 368). At the time of the last follow-up, 232 (63%) patients were compliant, 31 (8%) declined, and information on compliance with endocrine therapy could not be obtained in 105 (29%) patients. Regarding the distribution of the type of endocrine treatment being used the following medications were documented: aromatase inhibitor (153 postmenopausal pts, 4 premenopausal pts.), Tamoxifen (23 postmenopausal pts., 81 premenopausal pts.), various endocrine medications (25 postmenopausal pts., 27 premenopausal pts.), none (29 postmenopausal pts., 7 premenopausal pts.), and no data (10 postmenopausal pts., 3 premenopausal pts.).

Of the 123 patients to whom adjuvant chemotherapy was recommended, 89 (72%) were compliant, whereas 34 (28%) refused. One out of the 245 patients (0.4%) to whom adjuvant chemotherapy was not recommended underwent chemotherapy without tumor board recommendation. Overall, of the 130 EPclin high-risk patients, 87 (66.9%) underwent chemotherapy, whereas 30 (23.1%) patients opposed the recommended chemotherapy and 13 (10.0%) did not receive chemotherapy following tumor board recommendation. Among the high-risk patients to whom chemotherapy was recommended, the non-compliant patients (*n* = 30) tended to be older, have less nodal involvement and more G1 tumors, compared to the compliant patients (*n* = 87), while the median EPclin score was identical between the two subgroups (Table 2). Of the 238 EPclin low-risk patients, three (1.3%) underwent chemotherapy, whereas 235 (98.7%) did not.

### Survival analysis

**Fig. 3:**
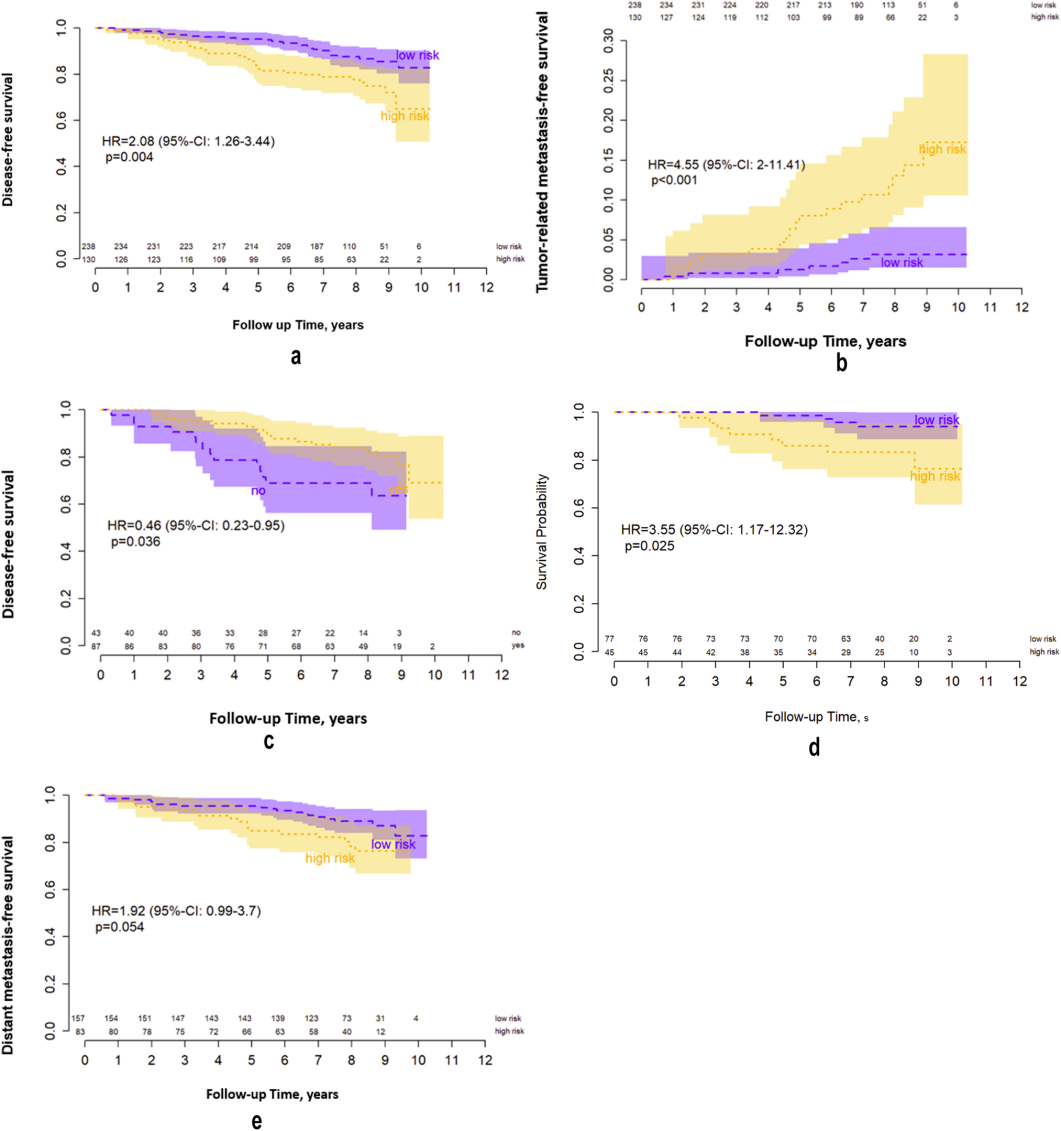
Survival analysis. **a** Disease-free survival by EPclin risk category. **b** Tumor-related metastasis-free survival by EPcln risk category. **c** Disease-free survival in EPclin high-risk patients according to receipt of chemotherapy. **d** Distant metastasis-free survival by EPclin risk category in premenopausal patients. **e** Distant metastasis-free survival by EPclin risk category in postmenopausal patients

Median follow-up was 8.2 (range 0.6–10.2) years. Five-year DFS was 95.3% (95% CI 92.6–98.0%) in the EPclin low-risk group versus 82.4% (95% CI 75.9–89.3%) in the EPclin high-risk group. With a HR of 2.08 (95% CI 1.26–3.44; *p* = 0.004), risk for disease recurrence or death in EPclin high-risk patients was twofold higher than in the EPclin low-risk patients (Fig. 3a). The 5-year DMFS in the EPclin low-risk group was 96.6% (95% CI 94.3–98.9%) and 85.5% (95% CI 79.6–92.0%) in the EPclin high-risk group. With a HR of 2.21 (95% CI 1.27–3.88; *p* = 0.005), the risk for distant metastasis or death in EPclin high-risk patients was more than twofold higher in comparison with EPclin low-risk patients. When considering only cancer-related death or distant recurrence EPclin high-risk patients were at significant higher risk than EPclin low-risk patients (HR 4.55; 95% CI 2.00–11.41; *p* < 0.001) (Fig. 3b).

The analysis of DFS in the EPclin high-risk patients according to receipt of adjuvant chemotherapy showed a significant benefit toward the patients receiving chemotherapy (HR 0.46; 95% CI 0.23–0.95; *p* = 0.036). The 5-year DFS for the high-risk patients who received chemotherapy was 89.1% (95% CI 82.7–96.1%) versus 68.9% (95% CI 56.2–84.5%) for those who did not (Fig. 3c). When focusing only on the high-risk patients who had a chemotherapy recommendation (*n* = 117), of whom 87 were compliant and 30 non-compliant, there was also a significant DFS benefit toward the patients receiving chemotherapy (HR 0.41; 95% CI 0.19–0.89; *p* = 0.025). Furthermore, in a Cox regression model corrected for age, nodal status, and tumor grade in this specific patient population (high-risk patients with chemotherapy recommendation), we also found DFS benefit toward patients receiving chemotherapy (HR 0.31; 95% CI 0.14–0.71; *p* = 0.007).

We also performed multivariable analyses of Epclin adjusted for age, nodal status, tumor grade, tumor size, and ki67. With these analyses the hazard ratio of the EPclin stratification is corrected for these factors showing a 2.5 fold higher risk of distant metastasis (MFS: HR = 2.5 (95% CI 0.29–327.61), *p* = 0.48) and a 2.6 fold higher risk of any breast cancer recurrence (DFS: HR = 2.6 (95% CI 0.32–338.8), *p* = 0.451) in the Epclin high-risk group. The given sample size was sufficient for consistent effect estimation but statistical significance could not be reached.

### Prognosis according to menopausal status

When considering subgroups according to menopausal status, EPclin high-risk patients were at significant higher risk of experiencing distant metastases or death than EPclin low-risk patients in both subgroups. In premenopausal patients, the 5-year DMFS was 98.6% (95% CI 96.0–100.0%) for the EPclin low-risk patients and 86.0% (95% CI 76.2–97.1%) for the EPclin high-risk patients (HR 3.55; 95% CI 1.17–12.32; *p* = 0.025) (Fig. 3d). In postmenopausal patients, 5-year DMFS was 95.5% for the EPclin low-risk patients (95% CI 92.3–98.8%) and 84.9% (95% CI 77.4–93.2%) for the EPclin high-risk patients (HR 1.92; 95% CI 0.99–3.70; *p* = 0.054) (Fig. 3e).

### Correlation between EPclin-based risk stratification and clinicopathological factors

**Fig. 4:**
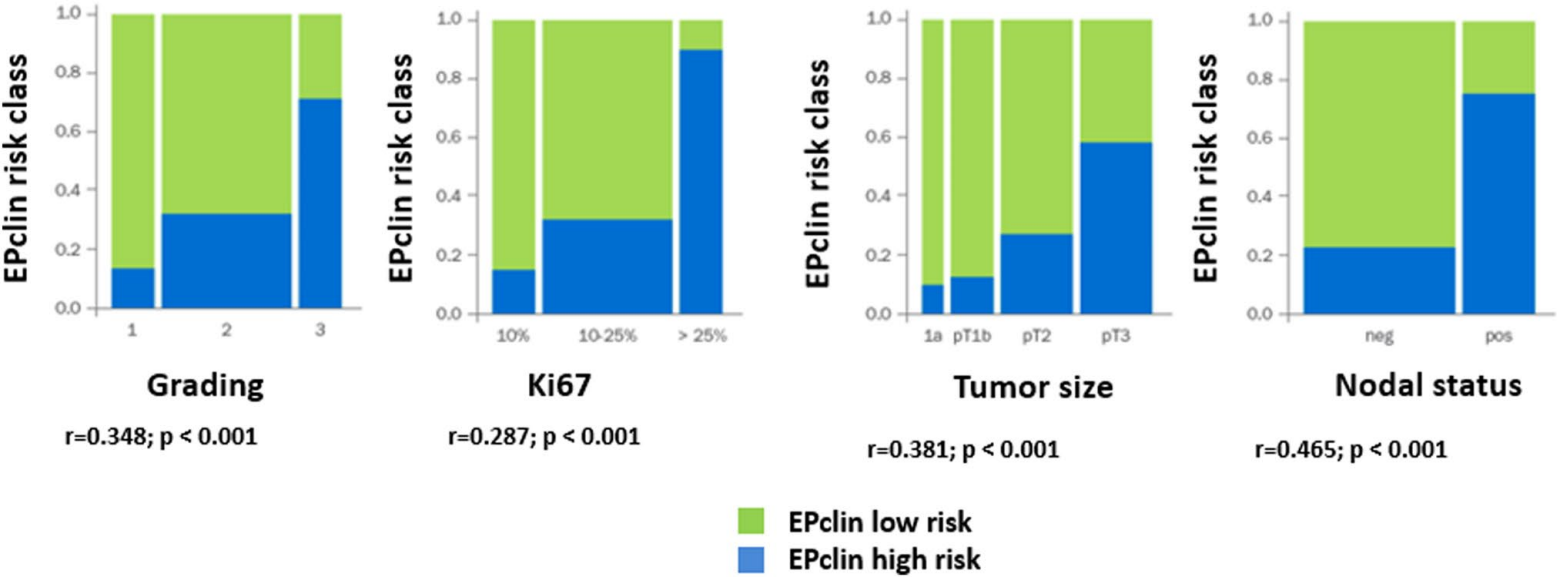
Correlation between EPclin risk class and tumor grade, Ki-67 expression levels, tumor size, and nodal status

We analyzed the effect of the EPclin classification in the context of Ki-67 subtypes. The determination of Ki-67 was possible in 306 (83.2%) samples. Ki-67 levels were low (< 10%; luminal A) in 73 (23.9%) patients, intermediate (10–25%) in 170 (55.6%) patients, and high (> 25%; luminal B) in 63 (20.6%) patients. We evaluated the correlation between EPclin risk class and tumor grade, Ki-67 distribution, tumor size, and nodal status (Fig. 4). There was moderate correlation (*r* = 0.465) with nodal status, and weak correlation with tumor grade (*r* = 0.348), Ki-67 (*r* = 0.287), and tumor size (*r* = 0.381). The EPclin-based low-risk classification was significantly associated with improved DFS compared to high-risk classification in both Ki-67 subtypes (Ki-67 low: HR 4.00; 95% CI 1.25–12.04; *p* = 0.021 and Ki-67 high: HR 3.77; 95% CI 1.19–18.93; *p* = 0.022). Using EndoPredict test result, 33.3% (21/63 patients) of all tumor samples classified as luminal B were re-classified toward the low-risk group, thereby sparing chemotherapy recommendation. On the contrary, 19.2% (14/73 patients) of all luminal A would be categorized to EPclin high-risk.

## Discussion

In this study, we report long-term prospective outcome data from a cohort of 368 patients whose adjuvant chemotherapy decision was based on risk stratification using EndoPredict. Our results confirm the early findings at 3-year follow-up [13], and show that risk stratification using EndoPredict allows the identification of high-risk patients who benefit from adjuvant chemotherapy in addition to endocrine therapy. We found a significant DFS benefit toward patients receiving chemotherapy when considering only EPclin high-risk patients who had a chemotherapy recommendation, also taking into account clinicopathological factors. It prospectively confirms a retrospective comparative analysis demonstrating the predictive power of EPclin [12].

Latest ASCO guidelines recommend the use of EndoPredict in postmenopausal women to guide decisions on adjuvant endocrine and chemotherapy [2]. Our results show that EndoPredict risk stratification is also applicable in premenopausal women. Our findings are consistent with those from a recent retrospective study that examined tumor samples from 385 premenopausal women with ER-positive, HER2-negative primary BC who did not receive chemotherapy [11]. The study showed that the EPclin score was associated with increased risk of distant recurrence within years of diagnosis (HR 3.58; 95% CI 2.26–5.66; *p* = 9.8 × 10^–8^). EPclin low-risk patients had a 10-year distant recurrence-free survival of 97% (95% CI 93–99%), compared to 76% (95% CI 67–82%; *p* = 0.0042) for EPclin high-risk patients. Interestingly, the findings in TAILORx and RxPONDER trials, which used Oncotype DX 21-gene recurrence score (RS) to stratify patients with ER-positive, HER2-negative, lymph node-negative (TAILORx) or node-positive with 1–3 positive nodes (RxPONDER) breast cancer, suggested that the performance and the used threshold of Oncotype DX depended on the menopausal status. The TAILORx trial demonstrated no chemotherapy benefit in patients with intermediate RS (11–25) aged > 50, although there was a benefit from adjuvant chemotherapy in patients with intermediate RS aged ≤ 50 [15]. This benefit in the premenopausal intermediate subgroup still remains unclear, as it might be due to chemotherapy-induced amenorrhea rather than direct cytotoxicity. Hopefully the ongoing NRG-BR009 OFSET trial will clarify this further. In the RxPONDER trial, postmenopausal patients with RS 0–25 did not benefit from adjuvant chemotherapy, whereas chemotherapy benefit was observed in premenopausal patients [16]. On the other hand, and in line with the data of Constantinidou et al. [11], our data suggest that the performance of EPclin is the same in pre- and postmenopausal patients and that the outcome of low-risk patients is excellent independent of menopausal status so that chemotherapy can be safely omitted.

The Ki-67 index is a measure of tumor proliferation, and the association between Ki-67 expression levels and both prognosis and prediction of treatment response has been extensively investigated. However, the variation in the interpretation of the Ki-67 index and the uncertainty in the optimal Ki-67 cut-off make the use of Ki-67 index for risk stratification controversial [17]. In our cohort, we found a large proportion of discordant cases where patients with low Ki-67 tumors had a EPclin high-risk (19%), or those with high Ki-67 tumors had a EPclin low-risk (33%). Although the cut-off values used were different, Jank et al. found in their cohort of 1,652 patients that 54% of low Ki-67 (≤ 10%) tumors had a EPclin high-risk score, and 26% of high Ki-67 (> 20%) tumors had a EPclin low-risk score [18]. Similarly, in a previous study, 29% of low Ki-67 (< 14%) tumors were re-classified as high-risk and 34% of high Ki-67 (> 14%) tumors were classified as low-risk according to EPclin [6]. In our cohort, the EPclin-based low-risk classification was significantly associated with improved DFS in both Ki-67 subtypes, indicating that EndoPredict provides a more exact estimation of prognosis than that provided by Ki-67 subtypes.

This study has several strengths. Firstly, it provides prospectively collected real-world evidence on the use of EndoPredict test to guide chemotherapy decision. Secondly, the subgroup analysis according to menopausal status provides much needed prospective data on the use of EndoPredict test in premenopausal patients. Finally, Ki-67 expression was centrally determined. The study’s limitations include the moderate sample size and its monocentric and non-randomized design. Follow-up will continue to collect data on late recurrences.

A large prospective multicenter non-interventional study on risk assessment by the EndoPredict test and long-term patient outcome in early luminal breast cancer (RESCUE: Reaching for evidence-based chemotherapy use in endocrine sensitive breast cancer—A prospective NCT03503799) has finished recruitment [19]. Survival data of patients who have been tested with EndoPredict are systematically assessed to prospectively prove that patients with a low-risk classification by EndoPredict can safely forgo chemotherapy and be treated with endocrine therapy alone. First results of the data enrollment will be reported in Q2/2024 and survival data will be published according to the planned analysis time points.

## Conclusion

These first long-term prospective outcome data provide real-world evidence and confirm that EndoPredict can guide decisions on adjuvant chemotherapy in early ER-positive, HER2-negative breast cancer. Patients categorized as EPclin high-risk benefited from adjuvant chemotherapy. Our results indicate that EndoPredict risk stratification also appears to be applicable in premenopausal women.

Furthermore, the EndoPredict test showed a better classification accuracy in comparison to Ki67 subtypes, resulting in a more precise estimation of prognosis.

## Data Availability

The individual patient data are legally restricted for publication because of legal matters with data privacy protection. Patients´ individual informed consent for publication was not given. Data requests may be sent to evelyn.klein@tum.de. This restriction is enforced by the data security office of Klinikum rechts der Isar, Technische Universität München (sekretariatdatenschutz@tum.de).
